# Epidemiological burden of multidrug-resistant bacteria and carbapenemases in Northern Morocco: a one-year observational study

**DOI:** 10.1186/s12879-026-13016-z

**Published:** 2026-03-14

**Authors:** Kawtar EL Harrak, Ikram AL Faqir, EL Mehdi EL Ghorba, Reda Amrani Souhli, Nouhaila Chahid, Moussaab Arbai, Majda El-Hassouni, Fadila Bousgheiri, Adil Najdi, Karima Rissoul

**Affiliations:** 1 Microbiology-Virology Laboratory, Mohammed VI University Hospital, Tangier, Morocco; 2 Department of Medical Biology, Faculty of Medicine and Pharmacy, Abdelmalek Essaadi University, Tangier, Morocco; 3 Epidemiology Laboratory, Faculty of Medicine and Pharmacy, Abdelmalek Essaadi University, Tangier, Morocco

**Keywords:** Multidrug-resistant bacteria, ESBL, Carbapenemase-producing *Enterobacterales*, Nosocomial infections, Antimicrobial resistance

## Abstract

**Introduction:**

Multidrug-resistant (MDR) bacteria represent a major public health challenge, contributing to therapeutic failure, increased healthcare costs, prolonged hospital stays, and higher mortality rates. This study aimed to investigate the epidemiology and resistance mechanisms of MDR bacteria in Northern Morocco in order to provide baseline data to support infection prevention and control strategies.

**Methods:**

This cross-sectional study was conducted throughout 2024 and included a wide range of clinical specimens processed in the microbiology laboratory of a tertiary-care university hospital. Bacterial identification was performed using matrix-assisted laser desorption ionization–time of flight (MALDI-TOF) mass spectrometry. Antimicrobial susceptibility testing (AST) was carried out using automated minimum inhibitory concentration (MIC) determination with the BD Phoenix™ M50 system and disk diffusion, with interpretation according to the 2021 CA-SFM/EUCAST guidelines. Carbapenemase genes were detected using multiplex PCR with the GeneXpert® Xpert Carba-R system (Cepheid, Sunnyvale, CA, USA).

**Results:**

A total of 4561 clinical samples were analyzed, of which 286 (6.27%; *N* = 286/4561) yielded MDR isolates. Nearly half of these isolates originated from intensive care units (48.3%; *N* = 138/286). The most frequent MDR-related infections were bloodstream infections (34.6%; *N* = 99/286), urinary tract infections (19.2%; *N* = 55/286), and pneumonia (17.5%; *N* = 50/286). The predominant MDR pathogens were extended-spectrum *β*-lactamase (ESBL)-producing *Enterobacterales* (42.3%; *N* = 121/286) and multidrug-resistant *Acinetobacter baumannii* (21.7%; *N* = 62/286), followed by carbapenemase-producing *Enterobacterales* (CPE) (13.3%; *N* = 38/286) and multidrug-resistant *Pseudomonas aeruginosa* (11.5%; *N* = 33/286). Methicillin-resistant *Staphylococcus aureus* (MRSA) (9.8%; *N* = 28/286) and vancomycin-resistant *Enterococcus faecium*/*faecalis* (VRE) (1.4%; *N* = 4/286) were also identified. Among CPE isolates, NDM (57.9%; *N* = 22/38) and OXA-48 (21.1%; *N* = 8/38) were the predominant carbapenemase genes, while (21.1%; *N* = 8/38) co-harbored both. No IMP, VIM, or KPC genes were detected.

**Conclusion:**

MDR organisms represent a substantial burden of nosocomial infections in this tertiary-care hospital setting, with limited therapeutic options. These findings highlight the importance of sustained antimicrobial stewardship, infection prevention measures, and robust surveillance systems to optimize patient management and limit the impact of antimicrobial resistance.

**Clinical trial registration:**

Not applicable.

## Introduction

Multidrug-resistant (MDR) bacteria are a critical public health concern, undermining the efficacy of antibiotic therapy and leading to increased rates of treatment failure. MDR organisms, defined as resistant to at least one agent in three or more antimicrobial classes, significantly complicate clinical management. Notable MDR pathogens include methicillin-resistant *Staphylococcus aureus* (MRSA), extended-spectrum *β*-lactamase (ESBL)-producing *Enterobacterales*, vancomycin-resistant* Enterococcus faecium / faecalis* (VRE), multidrug-resistant *Pseudomonas aeruginosa* (MDR-PA), and *Acinetobacter baumannii* (MDR-AB). Difficult-to-treat resistant (DTR) organisms, particularly carbapenemase-producing *Enterobacterales* (CPE), are of particular clinical concern because of their resistance to last-line agents such as carbapenems and newer *β*-lactam/*β*-lactamase inhibitor combinations. Their presence in hospital settings further narrows therapeutic options and facilitates the spread of healthcare-associated infections [1].

The World Health Organization (WHO) has identified antimicrobial resistance (AMR) as one of the most urgent global threats to human health; by 2050, AMR-related mortality is projected to exceed that of cancer [2].

In Morocco, the burden of MDR infections represents a major challenge in both healthcare and community settings, highlighting the need for strengthened infection control policies, surveillance systems, and multidisciplinary collaboration. Therefore, this study aimed to investigate the epidemiology and resistance profiles of MDR and DTR organisms in Northern Morocco to generate data that could inform locally adapted prevention and treatment strategies.

## Methods

This cross-sectional, descriptive, and analytical study was conducted from January 1 to December 31, 2024, in the microbiology laboratory of Mohammed VI University Hospital (CHU of Tangier), the only tertiary-care university hospital serving Northern Morocco. Only clinical samples analyzed in this laboratory during the study period were included. 

The study included hospital-acquired multidrug-resistant (MDR) strains such as methicillin-resistant *Staphylococcus aureus* (MRSA), extended-spectrum *β*-lactamase (ESBL)-producing and carbapenemase-producing *Enterobacterales* (CPE), vancomycin-resistant *Enterococcus faecium / faecalis* (VRE), and multidrug-resistant *Pseudomonas aeruginosa* and *Acinetobacter baumannii*. 

Positive clinical specimens for these pathogens, including, blood cultures, urine, respiratory samples (protected specimen brush, bronchoalveolar lavage, sputum), pus, catheter tips, and sterile fluids (cerebrospinal, pleural, pericardial, and synovial) were included, whereas screening swabs for colonization (nasal or rectal) were excluded. 

Bacterial identification was performed using matrix-assisted laser desorption ionization–time of flight (MALDI-TOF) mass spectrometry. Antimicrobial susceptibility testing (AST) was carried out using the BD Phoenix™ M50 automated system (Becton, Dickinson and Company, Sparks, MD, USA) and confirmed by disk diffusion, interpreted according to the 2021 recommendations of the French Society for Microbiology (CA-SFM/EUCAST). 

Extended-spectrum β-lactamase (ESBL) production in *Enterobacterales* was detected by the double-disk synergy test, considered a reference phenotypic method, which identifies the clavulanic acid–induced enhancement of β-lactam activity.

Carbapenem resistance was screened using ertapenem, imipenem, and meropenem. Strains with reduced susceptibility underwent phenotypic confirmation with the modified Hodge test, followed by multiplex polymerase chain reaction (PCR) using the GeneXpert® Xpert Carba-R system (Cepheid, Sunnyvale, CA, USA) to detect *blaKPC*, *blaNDM*, *blaVIM*, *blaOXA-48*, and *blaIMP* genes directly from bacterial colonies, following the manufacturer’s instructions. 

Methicillin resistance in *S. aureus* was determined by cefoxitin disk diffusion and confirmed by oxacillin minimum inhibitory concentration (MIC) using the BD Phoenix system.

Vancomycin resistance in *Enterococcus faecium */ faecalis was assessed by disk diffusion (5 μg) and MIC testing, with resistance defined as an MIC ≥ 8 mg/L and/oran inhibition zone < 12 mm with visible growth within the zone.

Multidrug resistance in *P. aeruginosa* and *A. baumannii* was defined as reduced susceptibility to at least one agent in three or more antimicrobial classes, including aminoglycosides, carbapenems, and fluoroquinolones. 

Demographic and clinical data including sex, age, hospital ward, clinical diagnosis, specimen type, and resistance profile, were extracted from the Elabs hospital information system, compiled in Microsoft Excel, and analyzed using SPSS version 27(IBM Corp., Armonk, NY, USA). Categorical variables were expressed as frequencies and percentages, and continuous variables as means ± standard deviations. Associations were tested using the chi-square (χ2) test or Fisher’s exact test when expected cell counts were <5. P-values are reported as exact two-sided values, with statistical significance set at p < 0.05 (p < 0.001 when appropriate). 

## Results

Among the 4561 clinical specimens analyzed, 286 (6.27%; *N* = 286/4561) yielded multidrug-resistant (MDR) bacteria. The male-to-female ratio was 1.28, and the mean age of patients was 38.5 ± 26.6 years. Most isolates originated from medical and surgical intensive care units, which accounted for (48.3%; *N* = 138/286) of MDR cases. The most frequent infections caused by MDR organisms were bloodstream infections (34.6%; *N* = 99/286), urinary tract infections (19.2%; *N* = 55/286), pneumonia (17.5%; *N* = 50/286), purulent infections (13.3%; *N* = 38/286), and device-related infections (10.2%; *N* = 29/286) (Table 1).

**Table 1:** Distribution of MDR isolates by sex, department, and specimen type

Variable		Number (*N* = 286)	Percentage (%)
Sex	Male	161	56.3
	Female	125	43.7
Department	Medicine	53	18.5
	Surgery	41	14.3
	Emergency	54	18.9
	Intensive Care	138	48.3
Specimen Type	Urine Culture	55	19.2
	Blood Culture	99	34.6
	Device-related	29	10.2
	Respiratory (PSB)	50	17.5
	Pus	38	13.3
	Other	15	5.2

**Fig. 1:**
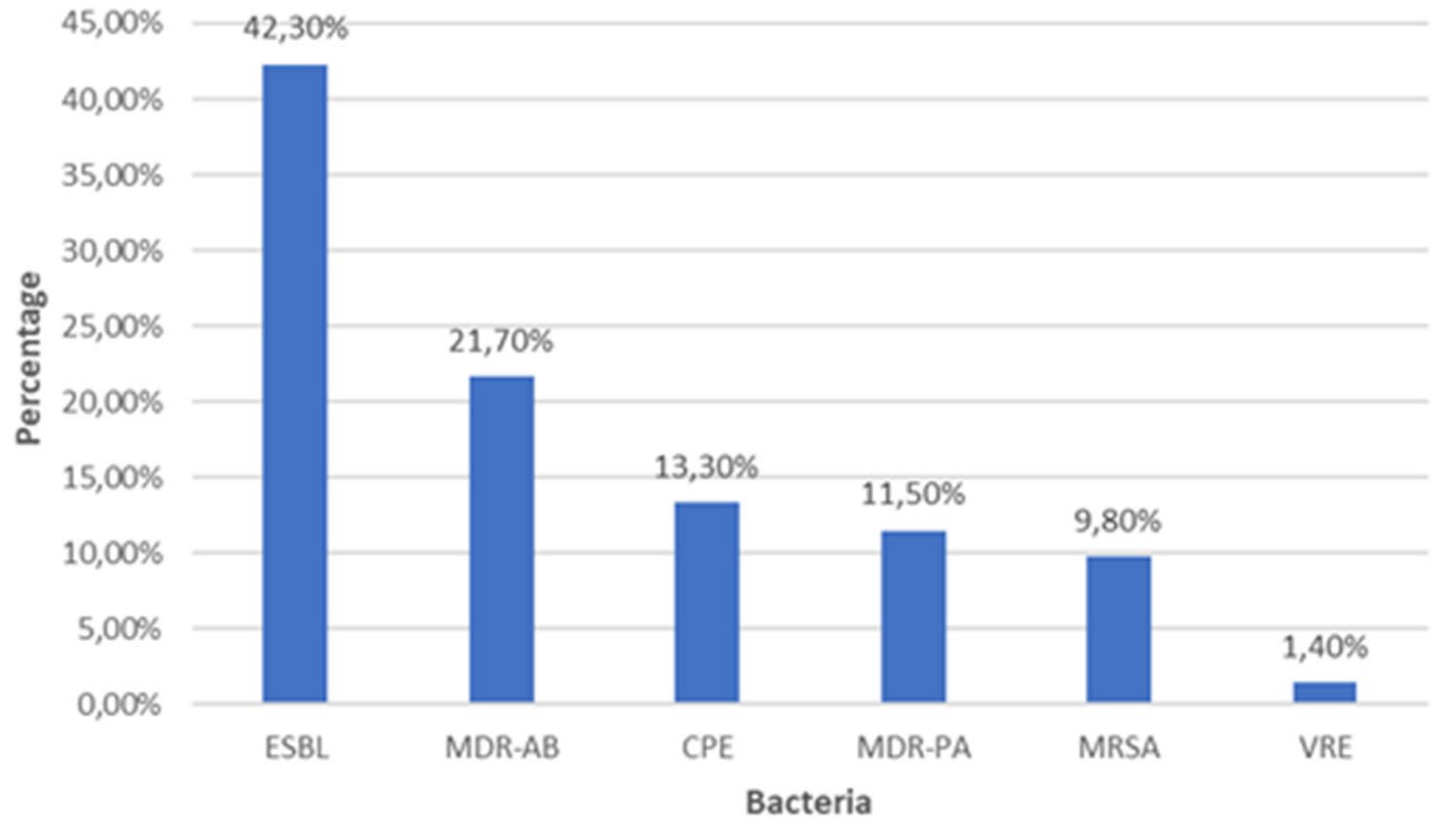
Composition of multidrug-resistant bacteria isolated

Extended-spectrum *β*-lactamase (ESBL)-producing *Enterobacterales* were the most frequently isolated MDR group, representing (42.3%; *N* = 121/286) of cases. Within this group, *Klebsiella pneumoniae* (55.4%; *N* = 67/121) and *Escherichia coli* (39.7%; *N* = 48/121) predominated, followed by isolated cases of *Klebsiella oxytoca*, *Citrobacter freundii*, *Citrobacter koseri*, and *Serratia marcescens*. Carbapenemase-producing *Enterobacterales* (CPE) accounted for (13.3%; *N* = 38/286), including 29 *K. pneumoniae*, 6 *E. coli*, and one isolate each of *C. freundii*, *Enterobacter cloacae*, and *K. oxytoca*. Methicillin-resistant *Staphylococcus aureus* (MRSA) represented (9.8%; *N* = 28/286) of MDR isolates, while vancomycin-resistant *Enterococcus faecium / faecalis* (VRE) were less frequent, accounting for (1.4%; *N* = 4/286), divided equally between *E. faecium* and *E. faecalis*. Multidrug-resistant *Acinetobacter baumannii* (MDR-AB) accounted for (21.7%; *N* = 62/286), and multidrug-resistant *Pseudomonas aeruginosa* (MDR-PA) for (11.5%; *N* = 33/286) (Fig. 1).

The distribution of multidrug-resistant bacteria by species is detailed in Table 2.

**Table 2:** Distribution of MDR isolates by bacterial species

MDR Isolates	Species	*n*	T	%
ESBL-producing *Enterobacterales*	*Klebsiella pneumoniae*	67	121	42.3
	*Escherichia coli*	48		
	*Klebsiella oxytoca*	3		
	*Citrobacter freundii*	1		
	*Citrobacter koseri*	1		
	*Serratia marcescens*	1		
Carbapenemase-producing *Enterobacterales*	*Klebsiella pneumoniae*	29	38	13.3
	*Escherichia coli*	6		
	*Citrobacter freundii*	1		
	*Enterobacter cloacae*	1		
	*Klebsiella oxytoca*	1		
Methicillin-resistant	*Staphylococcus aureus*	28	28	9.8
Vancomycin-resistant Enterococci	*Enterococcus faecium*	2	4	1.4
	*Enterococcus faecalis*	2		
Multidrug-resistant *Acinetobacter baumannii*		62	62	21.7
Multidrug-resistant *Pseudomonas aeruginosa*		33	33	11.5
**TOTAL MDR**		**286**	**286**	**100**

*Abbreviations*: *n*: number; T: Total per group; %: Percentage

A significant association was found between ESBL-producing isolates and medical and surgical wards (*p* < 0.001), while CPE were more frequently observed in intensive care units; however, this association was not statistically significant (*p* = 0.33). MDR-AB isolates were significantly more prevalent in intensive care and emergency departments (*p* < 0.001), suggesting strong selective antibiotic pressure in these settings (Table 3).

**Table 3:** Frequency of Multidrug-Resistant Bacteria (MDR) by department

Department	ESBL	CPE	MRSA	VRE	MDR-AB	MDR-PA
Medicine	62.3	13.2	9.4	1.9	1.9	11.3
Surgery	61.0	9.8	17.1	2.4	4.9	4.9
Emergency	25.9	7.4	9.3	3.7	33.3	20.4
Intensive Care	35.5	16.7	8.0	0.0	29.7	10.1
**P-value**	<0.001	0.33	NA	NA	<0.001	NA

*Note:* All values are presented as percentages (%)

**Fig. 2:**
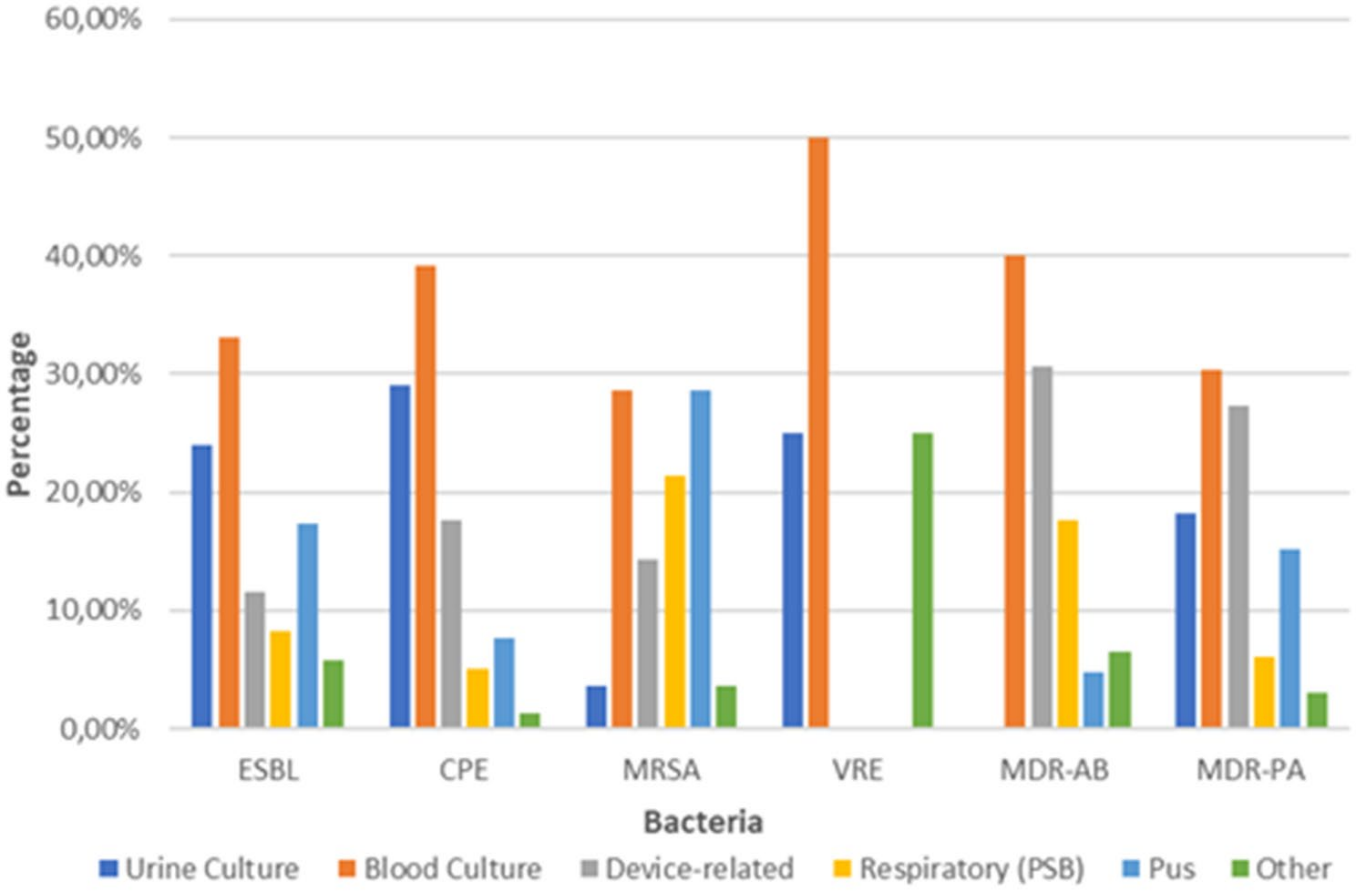
Distribution of MDR bacteria by specimen type

The distribution of MDR bacteria also varied by specimen type. ESBL and CPE isolates were predominantly detected in blood and urine cultures (*p* < 0.032), reflecting their involvement in bloodstream and urinary tract infections. MRSA isolates were mainly found in pus and blood cultures, whereas *P. aeruginosa* and *A. baumannii* were mostly isolated from respiratory samples and medical devices, confirming their role in healthcare-associated pneumonia and device-related infections (Fig. 2).

**Fig. 3:**
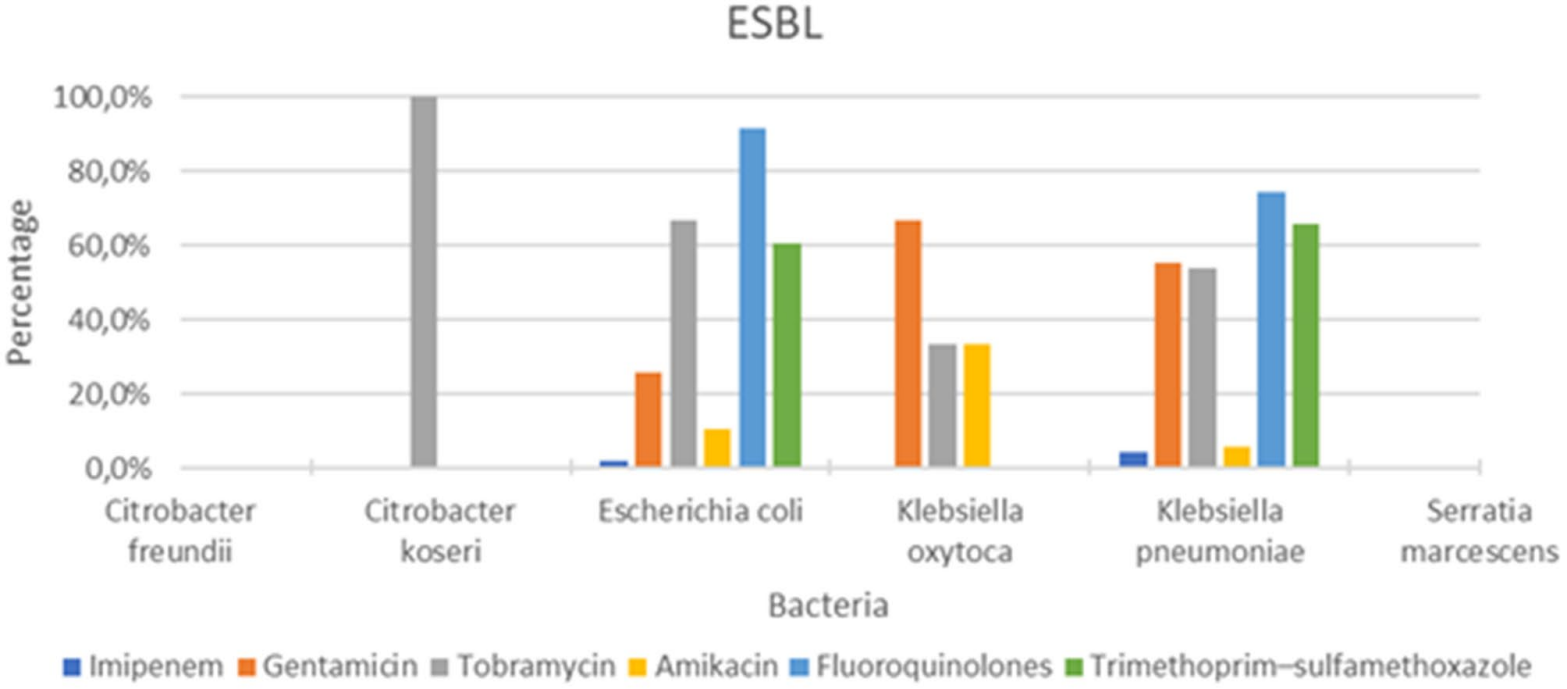
Resistance profile of ESBL-producing *Enterobacterales*

Regarding antimicrobial resistance profiles, ESBL-producing *K. pneumoniae* exhibited high resistance to fluoroquinolones (74%; *N* = 50/67), gentamicin (55%; *N* = 37/67), tobramycin (54%; *N* = 36/67), and trimethoprim–sulfamethoxazole (56%; *N* = 38/67), but remained largely susceptible to amikacin (6%; *N* = 4/67 resistant) and imipenem (4.5%; *N* = 3/67 resistant). ESBL-producing *E. coli* isolates showed resistance to fluoroquinolones (91.5%; *N* = 44/48) and trimethoprim–sulfamethoxazole (60%; *N* = 29/48), while resistance to amikacin (10.4%; *N* = 5/48) and imipenem (2%; *N* = 1/48) remained low (Fig. 3).

**Fig. 4:**
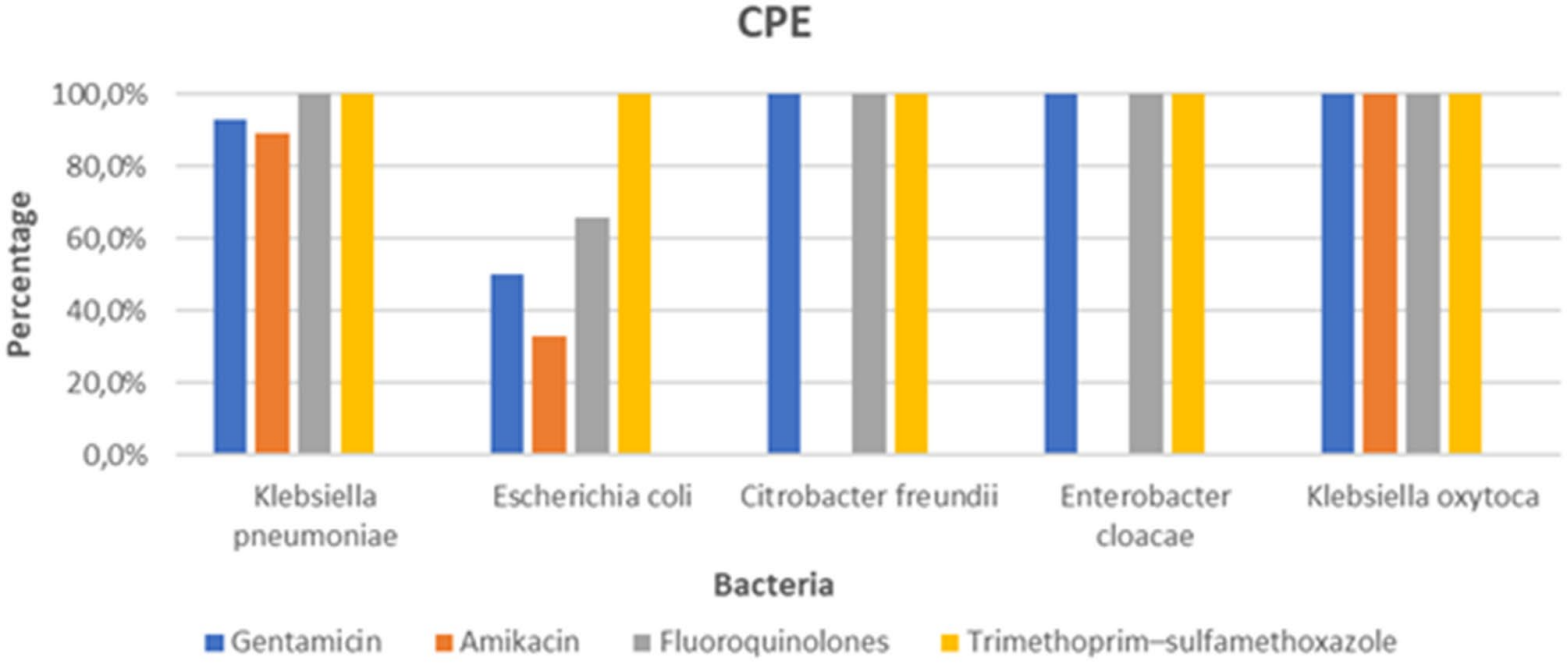
Resistance profile of carbapenemase-producing *Enterobacterales*

Carbapenemase-producing *K. pneumoniae* isolates were resistant to gentamicin (93%; *N* = 27/29), amikacin (89%; *N* = 26/29), and 100% to trimethoprim–sulfamethoxazole and fluoroquinolones. CPE *E. coli* isolates showed resistance to gentamicin (50%; *N* = 3/6), amikacin (33%; *N* = 2/6), fluoroquinolones (66%; *N* = 4/6), and 100% to trimethoprim–sulfamethoxazole (Fig. 4).

Among the 38 CPE isolates, the predominant carbapenemase genes detected were NDM (57.9%; *N* = 22/38) and OXA-48 (21.1%; *N* = 8/38), with (21.1%; *N* = 8/38) co-harboring both genes. No KPC, VIM, or IMP genes were detected. *K. pneumoniae* was the most frequent species (76.4%; *N* = 29/38), followed by *E. coli* (15.8%; *N* = 6/38), while *E. cloacae*, *C. freundii*, and *K. oxytoca* were each detected in one case (Table 4).

**Table 4:** Prevalence of carbapenemase genes among isolated strains

Species	*NDM*	*OXA-*48	*NDM *+ *OXA-*48
*Klebsiella pneumoniae* (*n* = 29)	19	3	7
*Escherichia coli* (*n* = 6)	2	3	1
*Citrobacter freundii* (*n* = 1)	0	1	0
*Enterobacter cloacae* (*n* = 1)	0	1	0
*Klebsiella oxytoca* (*n* = 1)	1	0	0

**Fig. 5:**
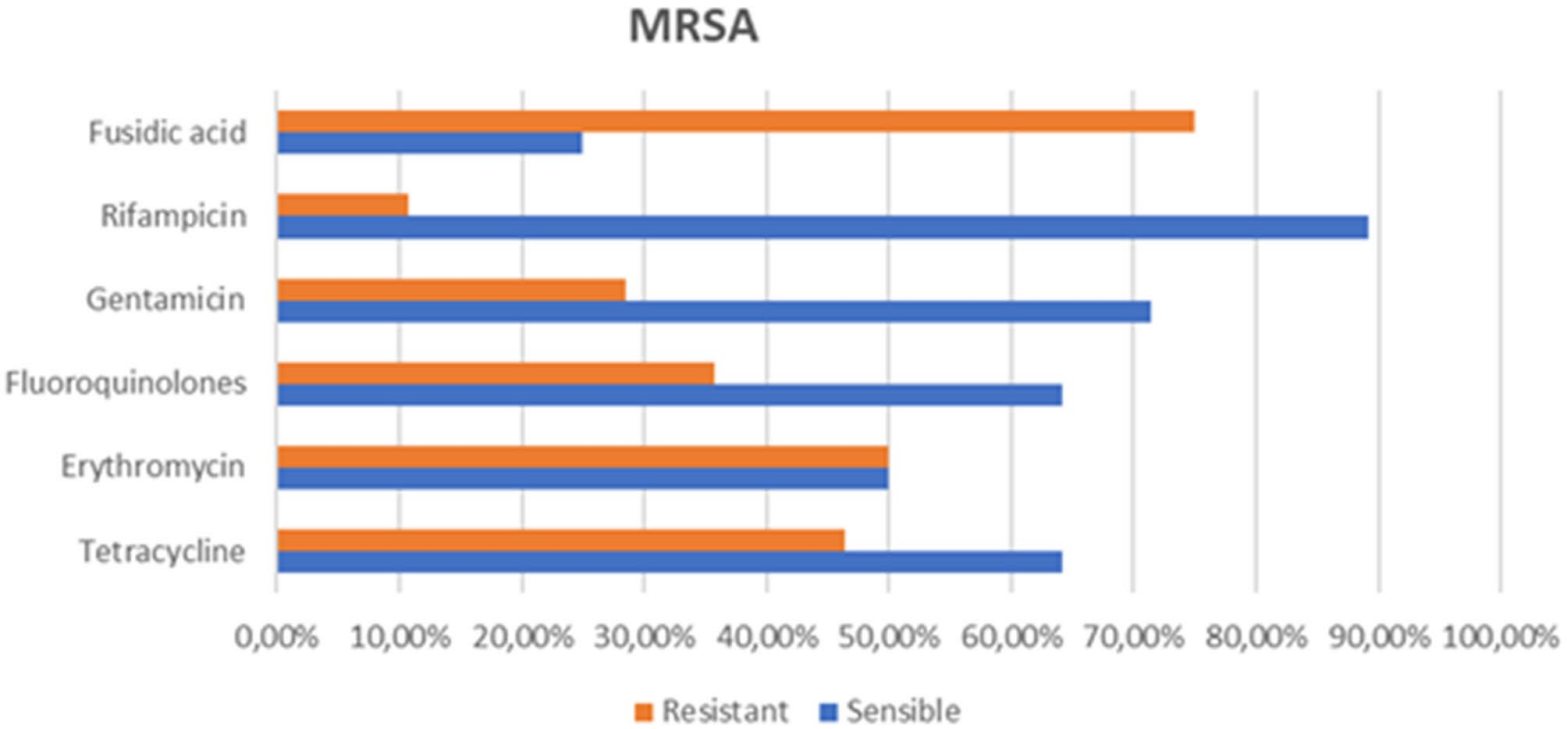
Resistance profile of methicillin-resistant Staphylococcus aureus

MRSA isolates demonstrated resistance to erythromycin (50.0%; *N* = 14/28), gentamicin (28.5%; *N* = 8/28), fluoroquinolones (35.7%; *N* = 10/28), tetracyclines (46.4%; *N* = 13/28), fusidic acid (75.0%; *N* = 21/28), and rifampicin (10.7%; *N* = 3/28) (Fig. 5).

VRE isolates exhibited complete resistance to ampicillin, aminoglycosides, fluoroquinolones, and teicoplanin, with one *E. faecalis* isolate (*N* = 1/4) resistant to linezolid.

All *A. baumannii* isolates displayed 100% resistance to third-generation cephalosporins, aminoglycosides, fluoroquinolones, and imipenem, confirming their multidrug-resistant profile. Similarly, *P. aeruginosa* isolates showed 100% resistance to ticarcillin–clavulanate, piperacillin–tazobactam, imipenem, amikacin, gentamicin, and fluoroquinolones, and (84%; *N* = 28/33) resistance to aztreonam.

**Fig. 6:**
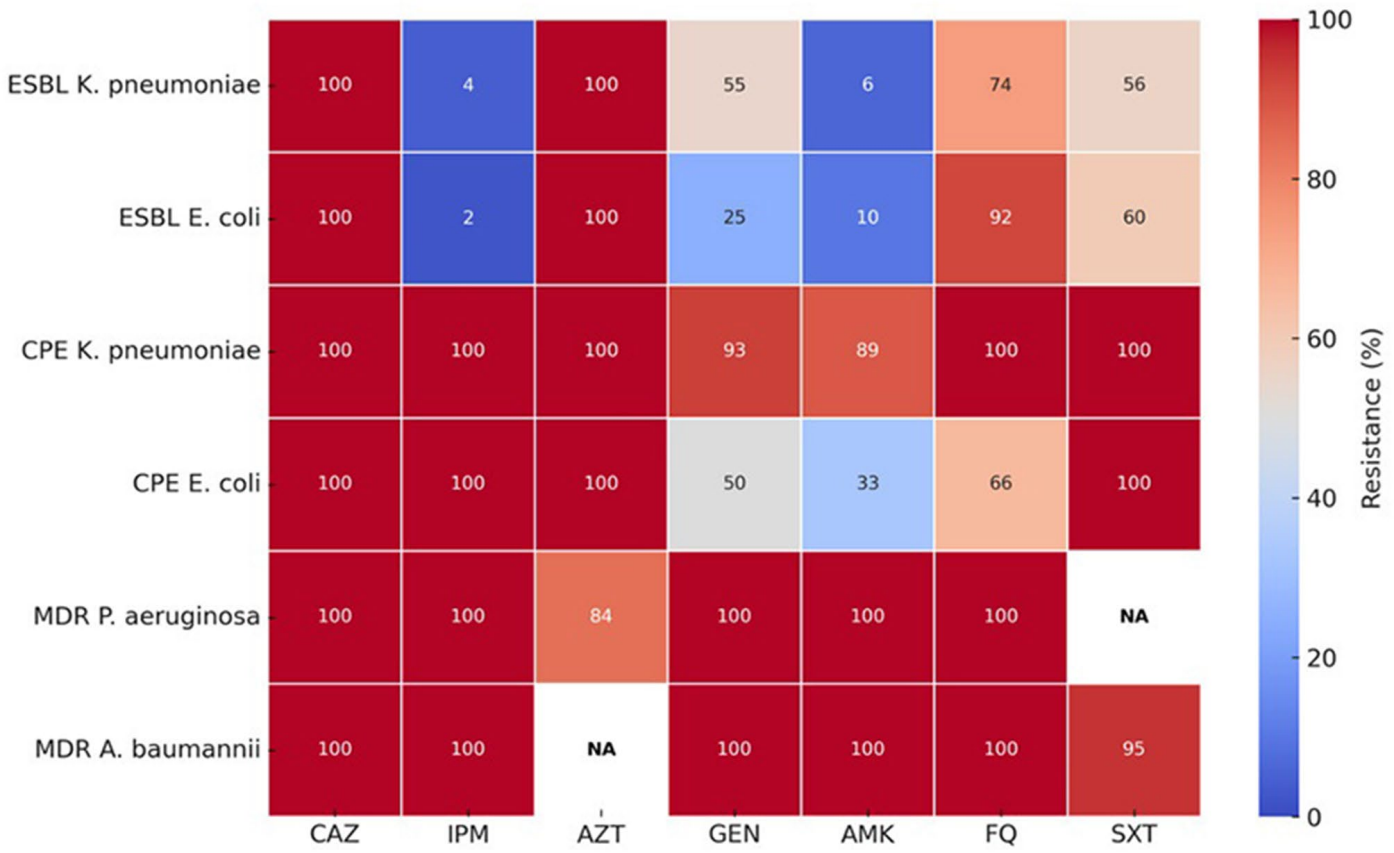
Gradient map of resistance rates (%) among major multidrug-resistant bacteria isolated at the University Hospital of Tangier, interpreted according to EUCAST 2021. Abbreviations: CAZ: ceftazidime; IPM: imipenem; AZT: aztreonam; GEN: gentamicin; AMK: amikacin; FQ: fluoroquinolones; SXT: trimethoprim–sulfamethoxazole; NA: not applicable (antibiotic not tested for this species)

The heatmap in Fig. 6 illustrates the marked contrasts in resistance between ESBL- and carbapenemase-producing *K. pneumoniae* and *E. coli*, as well as between *Enterobacterales* and non-fermenting species.

While amikacin and imipenem retained partial activity against ESBL producers, resistance was extremely high among carbapenemase producers and non-fermenters. *K. pneumoniae* CPE isolates exhibited very high resistance rates (gentamicin 93%, amikacin 89%, fluoroquinolones and trimethoprim–sulfamethoxazole 100%), whereas *E. coli* CPE isolates showed lower but still substantial resistance (gentamicin 50%, amikacin 33%, fluoroquinolones 66%, trimethoprim–sulfamethoxazole 100%).

*MDR A. baumannii* isolates were resistant to all tested antibiotics, and *P. aeruginosa* showed 100% resistance to most agents (84% to aztreonam).

Overall, these results highlight the substantial burden of multidrug-resistant pathogens in this tertiary-care hospital in Northern Morocco, particularly in intensive care units, and underscore the need for sustained antimicrobial stewardship and infection control programs.

## Discussion

Comparing the prevalence rates of MDR bacteria from our work with other surveys remains challenging, as differences in the number of infection sites, countries, healthcare settings, and study periods may influence observed trends. Although our study spans a single year, it provides valuable insight into MDR prevalence in this tertiary-care hospital setting. Cross-study comparisons should nevertheless be interpreted cautiously, given differences in denominators (specimen, isolate, or patient-based), case mix, and breakpoint standards. Moreover, susceptibility interpretations may vary depending on the guidelines and breakpoint versions used; in our study, results were interpreted according to the 2021 CA-SFM/EUCAST recommendations, whereas some compared studies relied on earlier EUCAST or CLSI criteria, which may partly explain discrepancies in reported resistance rates.

In this study, the prevalence of MDR bacteria was 6.27%, whereas at the Ibn Tofail University Hospital in Marrakech, it reached 15% in 2019 [3]. This rate remains lower than those reported in various international studies, such as 10.9% in Senegal in 2007 [4], 9.03% in Tunisia in 2005 [5], 9.8% in Algeria in 2001 [6], and 14.8% in France in 2018 [7]. In comparison, a Chinese point-prevalence survey reported a 2.1% rate of healthcare-associated infections (HAIs) [8]. Although HAI rates and MDR prevalence are not directly comparable, this figure helps contextualize the overall nosocomial burden in that setting and underscores the marked heterogeneity of infection dynamics across regions. Consistent with this heterogeneity, reported MDR prevalences also vary widely across settings: 7.1% in Europe in 2010 [9], 3.2% in the United States in 2015 [10], 5.9% in Switzerland in 2017 [11], and 7.4% in Japan in 2018 [12].

The prevalence of ESBL-producing *Enterobacterales* shows significant global variations, with markedly higher rates in developing countries compared to developed regions. The prevalence of ESBLs in our study was 42.3%, a higher rate than that observed at the Mohammed V Military Training Hospital (HMIMV) in Rabat, which reached 13.65% in 2020 [13]. Meta-analyses have shown a high prevalence of ESBLs in developing countries, such as Egypt, where the ESBL rate was 60% in 2023 [14], 40% in Pakistan in 2016 [15], 49% in Ethiopia in 2019 [16], 34.6% in Nigeria in 2019 [17], 29% in Nepal in 2021 [18], and 42% in East African hospitals in 2015 [19]. In contrast, prevalence rates in developed countries like Germany and the United Kingdom reach up to 10% [20, 21]. The percentage of ESBLs followed the same trend in France: 3.7% in 2015, 2.8% in 2018, and 3.0% in 2019 [22]. In Australia, an ESBL rate of 7.7% was reported in E. coli isolates from bacteremia cases in 2021 [23].

In our study, *K. pneumoniae* was the most frequent ESBL-producing organism (55.4%), followed by *E. coli* (39.6%). In comparison, French data from 2016 indicate that *E. coli* was the main ESBL-producing species at 58%, followed by *K. pneumoniae* (25%) and *Enterobacter cloacae* (11%). In 2017 in France, according to the national prevalence survey of healthcare-associated infections, 15% of *Enterobacterales* were ESBL-producers, compared to 14% in 2012 [24].

ESBL-producing *Enterobacterales* in our study showed high resistance to fluoroquinolones (78.5%), trimethoprim-sulfamethoxazole (57.8%), and gentamicin (42.3%). However, resistance to imipenem (3.3%) and amikacin (8.26%) remained low. In comparison, French data from 2015 to 2017 reported frequent resistance to gentamicin (43%), amikacin (14%), and fluoroquinolones (69%), but high susceptibility to carbapenems (97% for ertapenem and 99% for imipenem), according to the National Observatory for the Epidemiology of Bacterial Resistance to Antibiotics (ONERBA) 2018 [24].

According to data from Maghreb and European MDR surveillance networks, ESBL-producing *Enterobacterales* strains generally remain highly resistant to all antibiotics except carbapenems [25–27]. However, the use of carbapenems to treat infections due to third-generation cephalosporin-resistant *Enterobacterales* (3GCR-E) has contributed to the selection of strains with decreased susceptibility and resistance to carbapenems.

Regarding carbapenem-resistant *Enterobacterales*, this study revealed a prevalence of 13.3%. Carbapenems are often considered last-resort antibiotics in intensive care units, mainly for treating severe broad-spectrum systemic infections, used either as monotherapy or in synergistic combinations, and sometimes as empirical treatment without bacteriological confirmation, which induces the selection of resistant mutants and the spread of carbapenemase-producing bacteria [28].

Our carbapenemase-producing *K. pneumoniae* and *Escherichia coli* isolates were MDR to the tested antibiotics, notably aminoglycosides, fluoroquinolones, and trimethoprim-sulfamethoxazole, with resistance rates reaching 100%. Notably, this high resistance was identified exclusively among our carbapenemase-producing *K. pneumoniae* isolates. In line with our findings, an Egyptian study on *K. pneumoniae* isolates responsible for catheter-associated urinary tract infections reported similar multidrug resistance rates (94.9%) and complete resistance to imipenem, while amikacin remained the most active antibiotic (42.4% resistance). These results, consistent with our observations, suggest a shared regional trend between Morocco and Egypt, characterized by the dissemination of carbapenemase-producing strains and the gradual reduction in the effectiveness of last-resort antibiotics across the North African region [29].

To better understand this regional dynamic, we analyzed the resistance genes involved in our collection of isolates. The main genes encoding carbapenemases in *Enterobacterales* identified in our study were NDM (57.9%), OXA-48 (21.1%), and strains co-possessing NDM and OXA-48 genes (21.1%). IMP, VIM, and KPC genes were not detected in any isolates. The majority of CPE were isolated from patients with prolonged hospital stays in various departments, mainly intensive care, indicating that most of these strains are of nosocomial origin. In contrast, the Mohammed VI University Hospital in Marrakech reported in 2021: 35% OXA-48, 24% NDM, 7% VIM, and 34% with an NDM–OXA-48 combination [30]. According to the e-SIN system, France observed a progressive decrease in the proportion of OXA-48 (67% in 2019, 65% in 2020, 62% in 2021, and 57% in 2022) and a continuous increase in NDM (19% in 2019, 25% in 2020, 28% in 2021, and 32% in 2022). KPC and VIM have remained between 4 and 5% since 2019 [31].

Carbapenem resistance in *Enterobacterales* is linked to the combination of several resistance mechanisms: overexpression of extended-spectrum *β*-lactamases with efflux pumps, decreased membrane permeability, and expression of carbapenem-hydrolyzing carbapenemases. In *Enterobacterales*, carbapenemases represent the most important resistance mechanism, as carbapenemase genes are mainly plasmid-encoded, associated with multidrug or pandrug resistance, and transferable, at least among *Enterobacterales*, making them potentially responsible for outbreaks [32].

Beyond their carbapenemase resistance determinants, the clinical distribution of ESBL- and CPE-producing strains deserves particular attention. Our analysis demonstrated a significant association between ESBL/CPE-producing isolates and specific specimen types, notably blood and urine cultures (*p* < 0.032). Although this association could partly reflect the higher baseline frequency of these specimens in routine clinical practice, this explanation alone is insufficient. The exhaustive nature of our sampling strategy, which prospectively included all consecutive clinical isolates collected over a 12-month period (January–December 2024), supports the representativeness and clinical relevance of these findings. Therefore, this distribution is unlikely to be an artifact of sampling bias and instead highlights the substantial involvement of ESBL- and CPE-producing organisms in both urinary tract and systemic infections.

In our analysis, the prevalence of Methicillin-Resistant *Staphylococcus aureus* (MRSA) was 9.8%. A similar rate was observed at the Mohammed V Military Training Hospital in Rabat, with 9.2% in 2016 [33]. Furthermore, at the Ibn Tofail University Hospital in Marrakech, the prevalence was 12% in 2010, then reached 30% in 2017 before decreasing to 15% in 2019 [34]. The prevalence of MRSA in invasive infections has significantly decreased in France. It dropped from 32% in 2000 to 16% in 2014, and further to 12% in 2020. This positive trend has also been observed at the European level and is mainly explained by improved antibiotic stewardship and the increased use of alcohol-based hand rubs for hand hygiene [35].

Regarding the resistance of MRSA to other antibiotics, our study revealed variable resistance rates: 50% of the studied strains were resistant to erythromycin, 28.5% to gentamicin, 35.7% to fluoroquinolones, 75% to fusidic acid, and 10.7% to rifampicin. In comparison, data from the Rabat University Hospital in 2015 reported 51.6% of strains resistant to erythromycin, 30% to aminoglycosides, 61.3% to fluoroquinolones, and 48.4% to fusidic acid, with no resistance to vancomycin or teicoplanin. The prevalence of MRSA remains low and has been steadily decreasing since 2010 at the Ibn Sina University Hospital. However, co-resistance of MRSA to other antibiotic classes remains concerning [36].

MRSA is associated with the modification of the antibiotic target through the acquisition of PBP2a (Penicillin-Binding Protein 2a), which has a very low affinity for methicillin-type penicillins and other *β*-lactams. This additional PBP is encoded by a highly conserved gene, *mecA*, located within a mobile genetic element known as the Staphylococcal Cassette Chromosome (*SCCmec*) integrated into the bacterial chromosome [37].

Our results showed that Vancomycin-resistant *Enterococcus faecium* and *Enterococcus faecalis*(VRE) represented 1.4% of MDR bacteria, with complete resistance to ampicillin and teicoplanin, as well as one linezolid-resistant strain. Furthermore, VRE infections were observed in patients with risk factors such as prior corticosteroid therapy, sepsis, and vancomycin use during hospitalization. Given the small sample size and limited study period, this figure does not allow for reliable comparison with other studies.

In Europe, according to data from the EARSS network (European Antimicrobial Resistance Surveillance System), the percentage of VRE showed a significant increase, rising from 8% in 2012 to 17.3% in 2018. In contrast, in the United States, the percentage of VRE reached 30% in 2019 [38]. An analysis conducted in France between 2006 and 2022 revealed that the majority of VRE strains belonged to *Enterococcus faecium* (91.8%–98.8%), followed by *Enterococcus faecalis* (0.8%–7.9%) [38]. The vancomycin-resistant strains found in our study included two *Enterococcus faecium* and two *Enterococcus faecalis*.

Parallel to VRE, there is an increasing concern regarding linezolid-resistant *Enterococci* (LRE) strains, recently described as carrying transferable resistance genes. In our study, one linezolid-resistant strain was isolated (1/4), compared to four strains identified in France in 2016. This recent spread of LRE could be linked to the increasing use of linezolid in therapy and co-selection of cross-resistance induced by the combined use of linezolid and phenicols in hospitals [38].

The prevalence of Multidrug-Resistant *Acinetobacter baumannii* (MDR-AB) in our study was 21.7%, making this bacterium the second most frequently isolated pathogen after ESBL-producers. This ranking aligns with data from the literature. Reported frequencies in different studies vary, with a rate of 28.6% in Algeria in 2014 and 6.7% in France, according to the 2014 ONERBA annual report [39]. MDR-AB outbreaks are frequently observed in intensive care units, particularly among patients receiving mechanical ventilation, intravenous or urinary catheters, invasive procedures, and broad-spectrum antibiotics [40]. In our study, 29.7% of MDR-AB isolates were found in intensive care units. This figure is significantly higher than those published in the international report on intensive care units, which reported rates of 19.2% in Asia, 17.1% in Eastern Europe, 14.8% in Africa, 13.8% in Central and South America, 5.6% in Western Europe, 4.4% in Oceania, and 3.7% in North America [41].

In the present study, 33.3% of multidrug-resistant *Acinetobacter baumannii* (MDR-AB) isolates were recovered from patients admitted through emergency departments. This finding reflects the role of emergency units as a major point of hospital entry rather than suggesting a direct role in the dissemination of multidrug-resistant organisms. The persistence of MDR-AB in hospital environments has been associated with its ability to survive on inert surfaces and to form biofilms, which may facilitate environmental persistence and prolonged circulation within healthcare facilities [42]. Emergency departments frequently receive patients from diverse healthcare pathways, including outpatient care units, clinics, and peripheral hospitals, some of whom may already be colonized at the time of admission, thereby contributing to the introduction of MDR strains into the hospital setting [43]. Antibiotic management in emergency settings remains challenging due to the need for rapid decision-making in the absence of immediate microbiological data. In addition, antimicrobial selective pressure related to the frequent use of broad-spectrum empirical therapies, particularly in severe clinical situations, may favor the persistence of multidrug-resistant strains within healthcare environments [43].

Previous studies, including one conducted at Grenoble University Hospital, reported that only 53.1% of antibiotic prescriptions in emergency departments were considered optimal, highlighting variability in prescribing practices and the inherent complexity of antimicrobial decision-making in this context [44].

MDR-AB isolates in our study showed 100% resistance to all tested antibiotics, including third-generation cephalosporins, aminoglycosides, fluoroquinolones, and imipenem. New antibiotic molecules, such as cefiderocol (a siderophore cephalosporin) and the combination of two *β*-lactamase inhibitors, durlobactam and sulbactam, offer new therapeutic options for infections caused by *A. baumannii*, thus avoiding the use of polymyxin-class antibiotics, which have a less favorable safety profile due to nephrotoxicity. In some studies, patients have also been treated with bacteriophage suspensions [45].

Several mechanisms can explain carbapenem resistance in *A. baumannii*, particularly enzymatic inactivation of carbapenems by carbapenemases, which is the most frequent mechanism [46]. The carbapenemases often involved are oxacillinases such as OXA-23, OXA-40, and OXA-58. This carbapenemase production is often associated with membrane impermeability [47, 48]. The involvement of natural or acquired efflux systems in MDR-AB is increasingly being studied [49, 50].

In our study, Multidrug-Resistant *Pseudomonas aeruginosa* (MDR-PA) represented 11.5% of isolates, ranking fourth among MDR pathogens. Egypt reported the highest rate at 75.6%. A modest prevalence was noted in Saudi Arabia (7.3%) and Qatar (8.1%) [51]. The resistance rate of *P. aeruginosa* to antibiotics has remained relatively stable in France, varying from 12.0% in 2015 to 11.0% in 2018. In Spain, it decreased from 14.2% in 2015 to 10.9% in 2018. Similarly, in Germany, resistance progressively declined from 7.9% in 2015 to 6.0% in 2018 [52].

*P. aeruginosa* isolates in our study showed total resistance to all tested antibiotics, notably piperacillin-tazobactam, amikacin, gentamicin, fluoroquinolones, and imipenem, with an 84% resistance rate to aztreonam. Studies conducted in Saudi Arabia, Egypt, Syria, Libya, and Lebanon reported similar high resistance levels for piperacillin-tazobactam, cephalosporins, imipenem, monobactams, aminoglycosides, and fluoroquinolones [51]. The acquired resistance of *P. aeruginosa* to antibiotics, in addition to its natural resistance and ability to form biofilms, further complicates the therapeutic management of infections caused by this bacterium [53, 54].

Several mechanisms are responsible for this acquired MDR, including membrane impermeability, enzymatic inactivation, target mutation, and active efflux [55]. A study conducted in Qatar demonstrated that the introduction of effective antimicrobial stewardship programs (ASP) in 2015 reduced the prevalence of MDR-PA from 9% to 5.46% over a three-year period [56].

This study has several limitations. First, the one-year observation period does not allow detection of possible seasonal variations or long-term trends in antimicrobial resistance. Second, clinical data were not available, preventing correlation between microbiological findings and patient outcomes. Third, for *Enterobacterales*, colistin was tested using the BD Phoenix system; however, due to the absence of broth microdilution, the only method validated by CA-SFM/EUCAST for this molecule, results were not interpreted. Therefore, we did not report colistin susceptibility for *Enterobacterales* to avoid overinterpretation based on non-validated methods. This limits the clinical interpretation of the impact of the identified multidrug-resistant bacteria. Fourth, for some MDR categories, such as vancomycin-resistant *Enterococci*, the sample size was too small to enable in-depth analysis.

## Conclusion

Combating multidrug-resistant (MDR) bacteria requires a coordinated and continuously adaptive approach that integrates infection prevention measures, optimized antimicrobial stewardship, and robust surveillance systems. Close collaboration between microbiologists, infection prevention specialists, and clinicians remains essential to support appropriate therapeutic decision-making and improve patient outcomes.

Newly developed antimicrobial agents, including cefiderocol, ceftazidime–avibactam, imipenem–relebactam, and durlobactam–sulbactam, have expanded available treatment options for infections caused by MDR Gram-negative bacteria. Their use should be guided by microbiological confirmation and local antimicrobial susceptibility data, emphasizing the importance of sustained and standardized resistance surveillance.

In parallel, the careful re-evaluation of selected older antimicrobials, such as fosfomycin, nitrofurantoin, and mecillinam, may remain relevant in targeted clinical contexts when supported by locally validated susceptibility profiles.

Finally, the integration of digital tools and artificial intelligence into antimicrobial resistance surveillance networks may enhance data analysis, support early detection of resistance patterns, and inform future antimicrobial development strategies.

## Data Availability

The datasets generated during and/or analyzed during the current study are available from the corresponding author on reasonable request.

## Abbreviations

AMR: Antimicrobial Resistance
BSI: Antimicrobial Susceptibility Testing
CA-SFM: Committee of the Antibiogram of the French Society for Microbiology
CPE: Carbapenemase-Producing Enterobacterales
DTR: Difficult-to-Treat Resistant
EARSS: European Antimicrobial Resistance Surveillance System
ECDC: European Centre for Disease Prevention and Control
ESBL: Extended-Spectrum Beta-Lactamase
EUCAST: European Committee on Antimicrobial Susceptibility Testing
ICU: Intensive Care Unit
IMP: Imipenem
KPC: Klebsiella pneumoniae carbapenemase
MALDI-TOF: Matrix-Assisted Laser Desorption Ionization-Time of Flight
MDR: Multidrug-Resistant
MDR-AB: Multidrug-Resistant Acinetobacter baumannii
MDR-PA: Multidrug-Resistant Pseudomonas aeruginosa
MRSA: Methicillin-Resistant Staphylococcus aureus
NDM: New Delhi Metallo-beta-lactamase
ONERBA: National Observatory of the Epidemiology of Bacterial Resistance to Antibiotics
PCR: Polymerase Chain Reaction
PSB: Protected Specimen Brush
SPSS: Statistical Package for the Social Sciences
VIM: Verona Integron-Mediated Metallo-beta-lactamase
VRE: Vancomycin-Resistant Enterococcus
WHO: World Health Organization
